# Catalytic pyrolysis of *Laminaria japonica *over nanoporous catalysts using Py-GC/MS

**DOI:** 10.1186/1556-276X-6-500

**Published:** 2011-08-18

**Authors:** Hyung Won Lee, Jong-Ki Jeon, Sung Hoon Park, Kwang-Eun Jeong, Ho-Jeong Chae, Young-Kwon Park

**Affiliations:** 1 Graduate School of Energy and Environmental System Engineering, University of Seoul, Seoul 130-743, South Korea; 2 Department of Chemical Engineering, Kongju National University, Cheonan 330-717, South Korea; 3 Department of Environmental Engineering, Sunchon National University, Suncheon 540-742, South Korea; 4 Green Chemistry Research Division, Korea Research Institute of Chemical Technology, Daejeon 305-600, South Korea; 5 School of Environmental Engineering, University of Seoul, Seoul 130-743, South Korea

**Keywords:** *Laminaria japonica*, hierarchical meso-MFI zeolite, Al-MCM-48, Py-GC/MS

## Abstract

The catalytic pyrolysis of *Laminaria japonica *was carried out over a hierarchical meso-MFI zeolite (Meso-MFI) and nanoporous Al-MCM-48 using pyrolysis gas chromatography/mass spectrometry (Py-GC/MS). The effect of the catalyst type on the product distribution and chemical composition of the bio-oil was examined using Py-GC/MS. The Meso-MFI exhibited a higher activity in deoxygenation and aromatization during the catalytic pyrolysis of *L. japonica*. Meanwhile, the catalytic activity of Al-MCM-48 was lower than that of Meso-MFI due to its weak acidity.

## Introduction

The importance of alternative energy development has increased rapidly due to high international crude oil price. Therefore, many studies have been reported about producing bioenergy using various biomasses [1-6]. Among them, seaweeds are attractive biomass for fuel production, with higher production rates than land biomass due to their high photosynthesis efficiency [5]. When cultivated in the sea, seaweeds do not require water, land, or fertilizers, which reduces the cost and energy input. Producing biofuels and utilizing seaweeds residues reduce greenhouse gas emissions, as long as such activities do not disturb the food supply and marine ecosystem. Pyrolysis is one option for processing biomass for the production of feedstock and fuel [1-6]. The bio-oils produced via seaweeds pyrolysis can be used as heating fuel, but the fuel quality is low due to its high oxygen content [5]. In terms of importance of seaweeds as a potential source of biofuel, investigation on upgrading of seaweed-derived bio-oil would be very necessary. Even though researches of catalytic upgrading of bio-oil from micro-algae such as *Botryococcus braunii*, *Chlorella*, *Chaetoceros*, *Dunaliella*, *Nannochloropsis*, and *Spirulina *have been reported [7], the study of upgrading of bio-oil from seaweed has hardly been considered. Among various seaweeds, *Laminaria japonica *is a representative brown seaweed in East Asia. For example, the annual production of *L. japonica *is estimated to be 58 kt/year on a dry basis in 2008 in Korea [5]. Therefore, the study of upgrading bio-oil from *L. japonica *is highly desirable.

To enhance the quality of bio-oil, catalytic pyrolysis over microporous zeolites and nanoporous catalysts has been known to be very promising methods [8-13]. For the catalytic pyrolysis of biomass, it is desirable to apply nanoporous catalysts such as MCM-48 whose pore sizes are around 2 to 6 nm rather than microporous zeolite whose pore size is below 1 nm because nanoporous catalysts are advantageous for the decomposition of high molecular weight species due to their large pore size [11-15]. Also, the highly acidic catalyst would be better due to its high cracking ability. It has been reported that the catalytic activity of zeolites in cracking of hydrocarbons or biomass is correlated with their acidity [16-20]. In both terms of pore size and acidity, the more recently developed hierarchical meso-MFI zeolites (Meso-MFI) are suggested to apply for the catalytic pyrolysis of biomass due to its characteristics of high acidity and nanopore size [9,10].

Pyrolysis gas chromatography/mass spectrometry (Py-GC/MS) technique is a powerful tool to allow the direct analysis of the pyrolytic products. The product distribution after the catalytic reaction can be compared to reveal the catalytic effects of different catalysts. Furthermore, the chromatographic peak area of a compound is considered to be linear with respect to its quantity, and the peak area percent with its content. If the masses of the biomass and catalyst were the same during each experiment, the corresponding chromatographic peak area percent can be compared to show the change in the relative content of the pyrolysis vapors [6,13].

In this study, catalytic pyrolysis of *L. japonica *was investigated over nanoporous catalysts such as Meso-MFI and Al-MCM-48 for the first time. Their catalytic activities were analyzed in terms of the catalytic acidity and pore size.

## Experimental

### Synthesis of catalyst

The MCM-48 was prepared using the following procedure [15]. First, to prepare pure MCM-48, 10.0 g of cetyltrimethylammonium bromide, 1.5 g of Brij-30, and 190.5 g of distilled water were mixed. After the mixture became transparent, 46.13 g of a sodium silicate solution (Na/Si = 0.5) was slowly added dropwise under stirring. The prepared solution was reacted in a 100°C oven for 48 h, removed, and allowed to cool. Then, its pH was adjusted to 10 using 50 wt.% acetic acid, and the solution again reacted for another 48 h. The pH adjusting process was repeated three times. The solution was then washed with distilled water, filtered, and dried in the oven for 24 h. This was followed by another washing with ethanol and filtering, and again dried for 24 h and baked at 550°C for 4 h. Aluminum incorporation into MCM-48 was performed using the post-synthetic grafting method [16]. Before baking, the prepared MCM-48 was introduced into a solution prepared by dissolving AlCl_3 _in 100 mL of ethanol, according to the desired Si/Al ratio, and then stirred for 24 h, washed with ethanol, filtered, dried for 24 h, and calcined for 4 h at 550°C.

A Meso-MFI with a Si/Al molar ratio of 20 was synthesized using a procedure described elsewhere [9,10]. An amphiphilic organosilane, [(3-trimethoxysilyl)propyl]hexadecyldimethylammonium chloride, was used as a nanopore-directing agent. The catalyst thus obtained was calcined, ion-exchanged with a 1.0 M ammonium nitrate solution at 80°C repeatedly (four times) to convert it into the NH_4_^+ ^form, and finally calcined again at 550°C to convert it into the H^+ ^form.

### Characterization of catalyst

The powder X-ray diffraction (XRD) patterns were determined by X-ray diffractometer (Rigaku D/MAX-III) using Cu-Kα radiation. The Brunauer, Emmett, and Teller (BET) surface area of the catalyst was measured using an ASAP 2010 apparatus (Micromeritics, Norcross, GA, USA). The catalyst sample was dried, with 0.3 g of the dried sample taken, and outgassing under vacuum for 5 h at 250°C using nitrogen as an adsorption gas at the temperature of liquid nitrogen. The nitrogen adsorption-desorption isotherms and BET surface area were then obtained. The surface acidity of the catalysts was measured using temperature programmed desorption of ammonia (NH_3_-TPD) employing a BEL-CAT TPD analyzer with a TCD detector (BEL Japan Inc., Osaka, Japan). The Si/Al ratio of catalyst was verified by inductively coupled plasma atomic emission spectrometry (ICP-AES, Spectro Ciros Vision, PECTRO Analytical Instruments, Kleve, Germany). For measurements, sample (50 mg) was dissolved with nitrohydrochloric acid (5 ml) using a microwave oven. The decomposed solution was transferred through filter paper into a 100-ml calibrated flask, and the volume was adjusted to 100 ml with ultra-pure water.

### Py-GC/MS analyses

A double-shot pyrolyzer (Py-2020iD, Frontier Laboratories Ltd., Koriyama, Fukushima, Japan), coupled directly to GC/MS, was used for identification of the catalytic cracking products. For the sample preparation, the *L. japonica *(2 mg) and catalyst (1 mg) were placed in a sample cup and then into a 500°C furnace under a He atmosphere. The gaseous species generated during the catalytic cracking were directly introduced into a GC inlet port (split ratio of 1/100) and onto a metal capillary column (Ultra ALLOY-5MS/HT; 5% diphenyl and 95% dimethylpolysiloxane, length 30 m, i.d. 0.25 mm, film thickness 0.5 μm, Frontier Laboratories Ltd.). To prevent condensation of products, the interface and inlet temperatures were both maintained at 300°C. The column temperature was programmed to change from 40°C (5 min) to 320°C (10 min), at a heating rate of 5°C/min. The temperature of the GC/MS interface was 280°C, with the MS operated in the EI mode at 70 eV. The program was run in the scanning range from 29 to 400 a.m.u. at a rate of 2 scans/s. The identification of peaks was performed using the NISTMS library, with the area percents calibrated to compare the catalytic performance for the formation of valuable aromatic compounds. The experiments were conducted at least three times for each catalyst to confirm the reproducibility of the reported procedures. The average values of the peak area and peak area percent as received were calculated for each identified product. For the noncatalytic pyrolysis, only the *L. japonica *(2 mg) was placed in a sample cup and the same procedure with catalytic pyrolysis was applied.

## Results and discussion

### Characterization of *L. japonica*

Table 1 shows the physicochemical properties of the *L. japonica*. The *L. japonica *contained higher ash content and possessed higher amounts of O, N, and S. This led to significantly lower HHVs than the land biomass (about 20 MJ/kg). Therefore, the catalytic dexoygenation process should be carried out to enhance the properties of bio-oil synthesized from *L. japonica*.

**Table 1:** Physicochemical properties of *L. japonica*

Proximate analysis (wt.%)				**Ultimate analysis (wt.%)**^**a**^					HHV (MJ/kg)
Water	Volatile matter	Fixed carbon	Ash	C	H	**O**^**b**^	N	S	
7.65	53.10	10.97	28.28	30.60	4.89	62.44	1.51	0.56	6.41

^a^On ash-free basis; ^b^by difference. HHV, higher heating value.

### Characterization of catalysts

**Figure 1:**
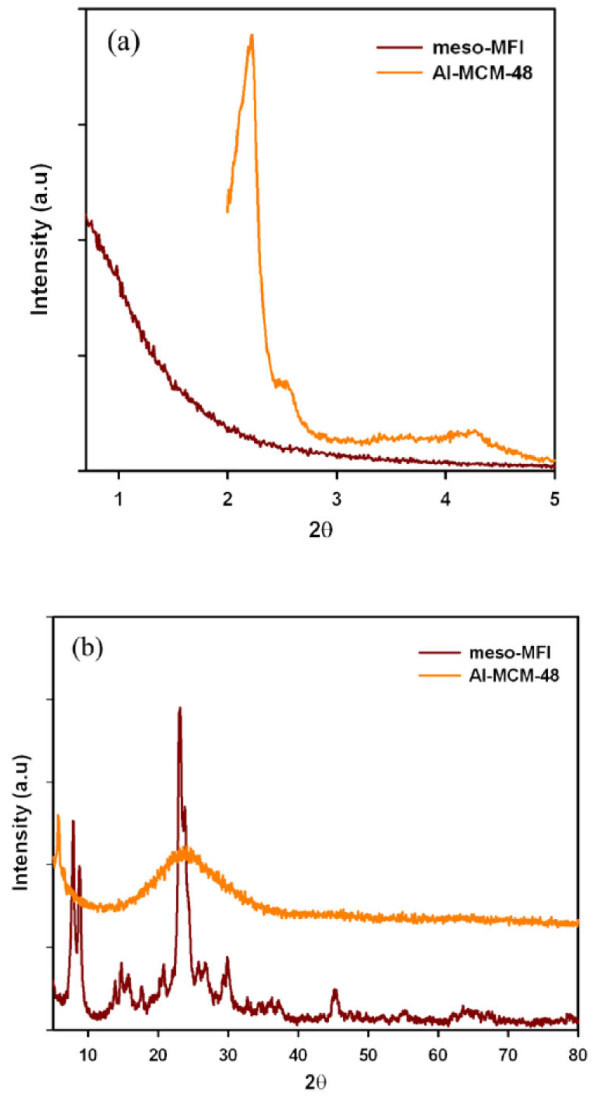
**XRD patterns of Al-MCM-48 and Meso-MFI catalysts (a) low angle (b) high angle**.

**Figure 2:**
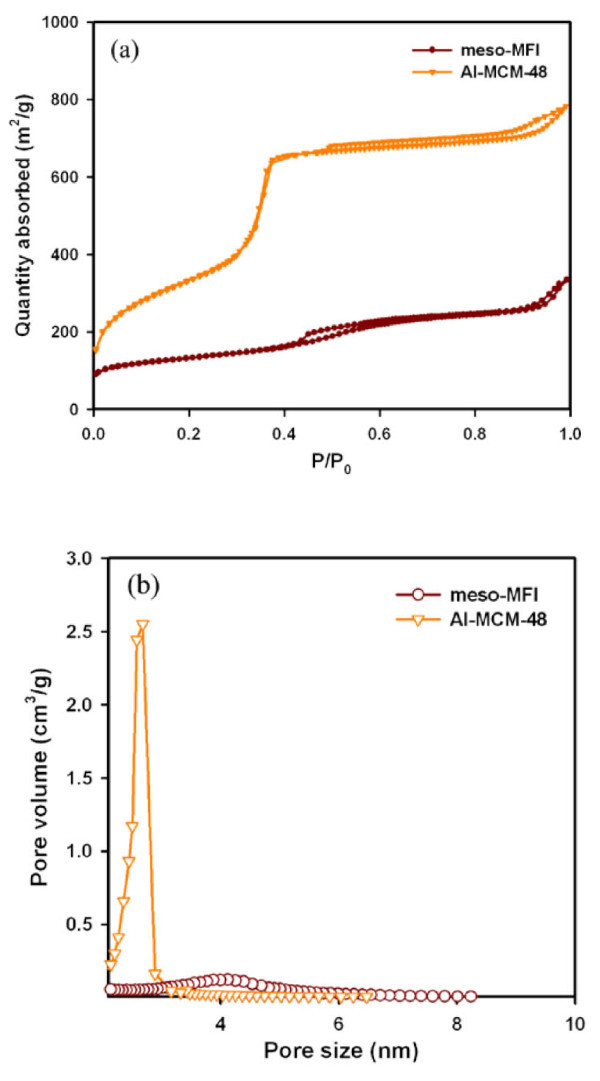
**Nitrogen adsorption-desorption isotherms (a) and pore size distributions (b) of nanoporous catalysts**.

As shown in Figure 1, the low angle of XRD pattern of Al-MCM-48 shows typical peaks of Al-MCM-48 and the high angle of XRD pattern of Meso-MFI is in accordance with the conventional MFI zeolite. Figure 2 exhibits the nitrogen adsorption-desorption isotherms and pore size distributions of the investigated catalysts. Both catalysts showed type IV isotherms in accordance with IUPAC classification. Al-MCM-48 exhibited an isotherm analogous to that of Al-MCM-41, a typical nanoporous material, whereas the Meso-MFI showed a slightly different isotherm from the hexagonal material with an increase in adsorption in the range of *P*/*P*_0 _= 0.8 to approximately 1.0. This was due to capillary condensation in the open mesopores [9,10], implying that the Meso-MFI had a greater textural porosity than Al-MCM-48.

Table 2 lists the textural properties of the catalysts. The BET surface area of Meso-MFI and Al-MCM-48 is 471 and 1, 219 cm^2^/g, respectively. The pore size of the Al-MCM-48 and Meso-MFI is 2.9 and 4.1 nm, respectively. Because the pore size of Meso-MFI is larger than that of Al-MCM-48, big molecules can be cracked into smaller molecules easily in Meso-MFI rather than Al-MCM-48. The Si/Al ratio of the catalysts was 20.

**Table 2:** Textural properties of nanoporous catalysts

Catalyst	**BET surface area (m**^**2**^**/g)**^**a**^	***V***_**p **_**(cm**^**3**^**/g)**^**b**^	**Average pore size (nm)**^**c**^	**Si/Al**^**d**^
Al-MCM-48	1, 219	1.21	2.6	20
Meso-MFI	471	0.51	4.1	20

^a^Calculated in the range of relative pressure (*P*/*P*_0_) = 0.05 - 0.20; ^b^measured at *P*/*P_0 _*= 0.99; ^c^mesopore diameter calculated by the BJH method; ^d^measured by ICP-AES. BET, Brunauer, Emmett, and Teller.

**Figure 3:**
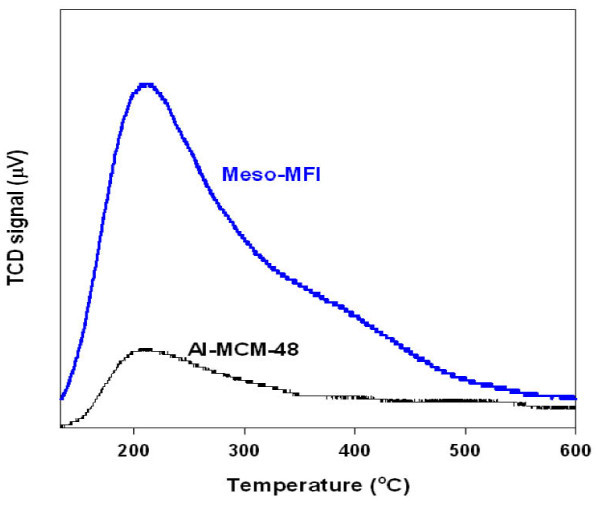
**NH_3 _TPD of Meso-MFI and Al-MCM-48**.

As shown in Figure 3, Al-MCM-48 has weak acidity because the peak at approximately 220°C was attributed to NH_3 _desorption from the weak acid. However, Meso-MFI showed two major peaks. The peaks at approximately 400°C was attributed to NH_3 _desorption from the strong Brönsted acid sites [9,10,17,18]. Also, the acid amount of Meso-MFI is higher than that of Al-MCM-48.

### Noncatalytic pyrolysis using Py-GC/MS

The bio-oil quality can be evaluated through the chemical composition [1-13]. Many researchers have classified the different bio-oil organic compounds into desirables, such as phenolics, alcohols, and hydrocarbons, and undesirables, such as acids, carbonyls, polycyclic aromatic hydrocarbons (PAHs), and heavier oxygenates [1-13]. Generally, these undesirable compounds should be removed because oxygenates such as carbonyls and acids are responsible for many side-reactions during storage. In addition, most PAHs are well-known toxic and mutagenic compounds, whereas mono aromatics, such as benzene, toluene, ethyl benzene, and xylenes, can be considered highly valuable chemicals due to their commercial applicability in the petrochemical industry. Also, phenolics are useful materials because it can be used for phenolic resin and petrochemicals.

**Figure 4:**
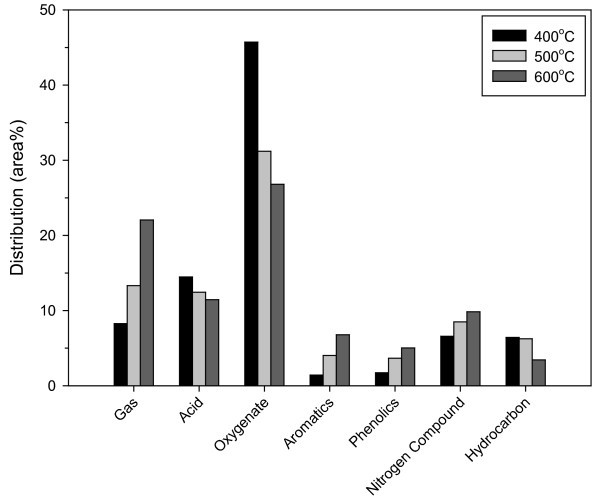
Product distributions obtained from pyrolysis of *L. japonica *at different temperatures

In this study, the pyrolysis products were roughly grouped into the following categories: gases (CO, CO_2_, and hydrocarbons up to C_4_), acids, oxygenates, aromatics, phenolics, nitrogen compounds, and hydrocarbons (aliphatic alkanes and alkenes). Figure 4 shows the chemical composition of the bio-oils obtained from *L. japonica *through noncatalytic pyrolysis at three different temperatures. With increasing temperature, oxygenates and acids were converted into other products such as phenolics, aromatics, and gases. This result implies that the bio-oil can be converted to high-quality fuels by pyrolysis at high temperature. However, a high-temperature cracking requires a lot of energy. Therefore, it would be better to make the same reaction take place at a lower temperature using catalysts.

### Catalytic pyrolysis

**Figure 5:**
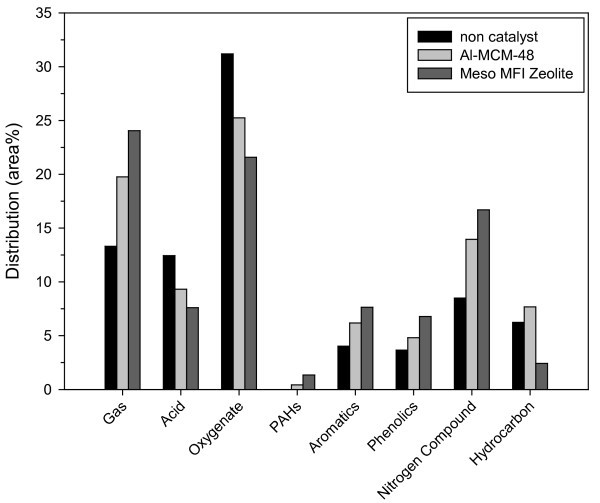
**Product distributions obtained from pyrolysis of *L. japonica *by catalytic pyrolysis at 500°C**.

Figure 5 shows the product distributions obtained from the pyrolysis of the *L. japonica*. Also, Table 3 shows the selected main components of bio-oil produced by pyrolysis at 500°C. Using the catalysts, the undesirable oxygenates and acids were reduced significantly. Meanwhile the valuable products such as aromatics and phenolics increased over nanoporous catalysts. It has been reported that synthesis of aromatics can be improved for the catalyst which has higher Brönsted acidity [9,10,17,18,21]. Strong acidic catalyst could accelerate the oligomerization of ethylene and propylene to form C_4_-C_10 _olefins, which then undergo dehydrogenation to form diolefins (or dienes). The subsequent cyclization and further dehydrogenation resulted in the formation of aromatic hydrocarbons.

**Table 3:** Selected main components of bio-oil produced by pyrolysis of *L. japonica*

Compound	Noncatalyst	Al-MCM-48	Meso-MFI
Acetic acid	3.44	4.36	4.17
Tetradecanoic acid	2.32	0.85	0.78
Z-7-Hexadecenoic acid	0.59	0.29	
*n*-Hexadecanoic acid	1.95	1.74	1.04
Octadecanoic acid	3.79	2.08	1.13
2-Cyclopenten-1-one, 2-methyl-	0.7	1.03	0.91
2-Cyclopenten-1-one, 3-methyl-	1.19	1.22	1.08
2-Cyclopenten-1-one, 2,3-dimethyl-	1.78	1.66	2.94
1,2-Cyclopentanedione, 3-methyl-	1.52	1.33	
2-Cyclopenten-1-one, 3-ethyl-	0.41	0.35	0.35
2-Cyclopenten-1-one, 3-ethyl-2-hydroxy-	0.86	0.85	0.57
Isosorbide	2.09	1.54	1.16
Naphthalene, 1,2-dihydro-3-methyl-		0.33	0.21
Naphthalene, 2-methyl-			0.32
Toluene	2.22	3.17	3.57
*o*-Xylene		1.03	1.48
Styrene	0.67	0.95	0.58
1H-Indene, 1-methyl-		0.41	0.81
1H-Indene, 1,1-dimethyl-		0.29	0.27
1H-Inden-1-one, 2,3-dihydro-	0.41	0.33	0.32
Phenol	0.87	1.44	1.85
Phenol, 2-methyl-	1.02	1.06	1.55
Phenol, 4-methyl-	1.01	1.27	1.39
Phenol, 3-ethyl-			0.61

In this study, more aromatic compounds were generated when Meso-MFI, which has strong Brönsted acid sites, was used compared to the case where Al-MCM-48 with weak acid sites was used. It can be suggested that some heavy compounds in the oil would react on the surface of the Meso-MFI catalyst and generate light hydrocarbons, such as ethylene and propylene. These light hydrocarbons then subsequently enter the pore of the Meso-MFI and undergo further polymerization and aromatization to form aromatic hydrocarbons. Also, similar results were reported from catalytic cracking over various catalysts: paraffinic hydrocarbons were the main products when nanoporous weak acidic Al-SBA-15 and Al-MCM-41 were used; whereas, the use of strong acidic HZSM-5 resulted in high yields of aromatic compounds [22]. In our results, Al-MCM-48 also produced higher hydrocarbon than Meso-MFI.

In addition, the high acidity could affect the production of gases [17,18]. Stronger acid sites can crack large molecules derived by thermal decomposition of *L. japonica *more easily, resulting in higher gas yields. Therefore, the use of strong acidic Meso-MFI resulted in a larger gas yield. Meanwhile, some amounts of undesirable PAHs due to its toxicity were produced for the catalytic upgrading. The high production of phenolics also may be ascribed to high acidity and large pore size of Meso-MFI. Heavy phenolics can be cracked into many small sizes of phenolics inside pore of Meso-MFI.

## Conclusions

Nanoporous catalysts, Meso-MFI and Al-MCM-48, were used for the catalytic pyrolysis of *L. japonica *using Py-GC/MS. Bio-oil was converted to valuable products over nanoporous catalysts. In particular, Meso-MFI showed higher catalytic decomposition ability than Al-MCM-48. Meso-MFI produced high yields of aromatics, phenolics, and gases due to its strong acidic sites which accelerate cracking of pyrolyzed bio-oil molecules.

## Abbreviations

Meso-MFI: meso-MFI zeolite; NH_3_-TPD: temperature programmed desorption of ammonia; Py-GC/MS: pyrolysis gas chromatography/mass spectrometry; XRD: X-ray diffraction.

## Competing interests

The authors declare that they have no competing interests.

## Authors' contributions

HYL, JKJ, SHP, KEJ, and HJC participated in some of the studies and participated in drafting the manuscript. YKP conceived the study and participated in all experiments of this study. Also, YKP prepared and approved the final manuscript.
